# Growth Behavior of Multi-Element Compound Layers During Reactive Diffusion Between Solid CoCrFeMnNi Alloy and Liquid Al

**DOI:** 10.3390/ma18174158

**Published:** 2025-09-04

**Authors:** Longtu Yang, Yufeng Yang, Zeqiang Yao, Shichao Liu, Yong Dong

**Affiliations:** 1 Innovation Research Institute of Low Carbon Metallurgical Engineering, School of Materials and Energy, Guangdong University of Technology, Guangzhou 510006, China; 2 School of Tron and Steel, Soochow University, Suzhou 215021, China; sc_liu@suda.edu.cn

**Keywords:** CoCrFeMnNi HEA, interdiffusion behavior, intermetallic compounds, diffusion mechanism, growth mechanism

## Abstract

In the present study, the diffusion couple of solid CoCrFeMnNi HEA and liquid pure Al was prepared. The microstructure evolution and relevant interdiffusion behavior of CoCrFeMnNi HEA/Al solid–liquid diffusion couple processed by different parameters were characterized and investigated. Results demonstrated that the interfacial compounds in the order of Al(Co, Cr, Fe, Mn, Ni), Al_13_(Co, Cr, Fe, Mn, Ni)_4_ and Al_4_(Co, Cr, Fe, Mn, Ni) were determined in the interdiffusion area along the direction from CoCrFeMnNi HEA to Al, and the precipitated Al_4_(Cr, Mn) and Al_9_(Co, Fe, Ni) phases were formed in the center of Al couple. In addition, the diffusion mechanism and activation energy of growth for each diffusion layer were revealed and determined. More importantly, the growth mechanism of each diffusion layer was also investigated and uncovered in detail. Meanwhile, the activation energy of each intermetallic layer was obtained by the Arrhenius equation and the linear regression method. It is anticipated that this present study would provide a fundamental understanding and theoretical basis for the high-entropy alloy CoCrFeMnNi HEA, potentially applied as the cast mold material for cast aluminum alloy.

## 1. Introduction

Cast aluminum alloy, possessing high specific strength and machinability, is widely used in aerospace, automotive, construction, machinery and food packaging industries, etc. [1,2,3,4,5,6,7]. However, during the cast process, the molten aluminum alloy would corrode the cast mold, thus reducing the reuse rate and life span of the cast mold. Moreover, the resultant corrosion of the cast mold would inevitably affect the quality of the workpiece [8]. In this regard, such applied materials for cast mold are required to possess high heat resistance and anti-corrosion properties [9]. In recent years, steel, graphite and gypsum are the most commonly used industrial cast mold materials for aluminum alloy [10,11,12,13,14]. For steel molds, even the surfaces are covered by chalk or sprayed with titanium dioxide; over extended periods of use and thermal cycling, degradation and diffusion through the coating can still lead to the eventual formation of a limited intermetallic layer. The formation of harmful intermetallic compounds resulting from the reaction between the steel mold and molten aluminum, which exhibits hard and brittle performance, would increase the risk of mold cracking and reduce the life span of the mold significantly [15,16,17,18,19]. Although the graphite mold shows high thermal shock resistance and thermal conductivity, such a mold would be seriously consumed by molten aluminum; accordingly, this obviously improves the production costs [10,11]. As for the gypsum mold, it has a significant, excellent economic advantage, which could fabricate complex cavities to fulfill the requirements of workpieces with sophisticated structures [12]. However, the downside is that the uneven temperature distribution in the gypsum mold due to the low thermal conductivity would result in the mold cracking without difficulty during the process of gel-hardening, thermal baking and casting. Given the above, the suitable mold material should ensure both its own sustainability and fulfill the needs of the workpiece.

A new type of design concept of alloys has been proposed by Yeh et al. and Cantor et al. in 2004 [20,21]. The alloys are named high-entropy alloys (HEAs) or multi-principal element alloys (MPEAs). It has been demonstrated that HEAs show a favorable and promising future in mold manufacturing through the reasonable utilization to increase productivity and cost savings. Different from traditional alloys, multi-principal elements of HEAs result in four effects, including the high entropy effect, lattice distortion effect, slow diffusion effect and cocktail effect [22]. They also exhibit excellent performance in both superconducting [23] and magnetic properties [24]. Given that, when applied as the aluminum cast mold material, the aluminum atoms diffuse, which dominates the formation of intermetallic compounds, would be hindered due to the lattice distortion effect and the slow diffusion effect. In this case, the reuse rate and life span would be increased due to the reduction of intermetallic compounds. On the other hand, HEAs could fulfill the needs of cast progress such as strength, corrosion resistance, thermal conductivity and machinable property benefited by high entropy effect and cocktail effect [25,26,27,28,29]. Provided that the Hume–Rothery principle is satisfied, the elemental composition of HEAs is diverse.

The CoCrFeMnNi HEA is noted for its remarkable mechanical properties over a wide range of temperatures [30,31]. Numerous research studies have been carried out focusing on the CoCrFeMnNi HEA, which exhibits the single-phase face-centered-cubic structure. The excellent performance of the CoCrFeMnNi HEA in abrasion resistance and thermal stability could qualify the production of aluminum cast mold [32,33]. At the same time, the production of CoCrFeMnNi HEA shows an advantage due to the ease of processing and equilibrium in strength and plasticity [34,35,36]. Based on current research on CoCrFeMnNi high-entropy alloys, it has been found that the phase structure and mechanical properties of such alloys can be modified by adjusting the aluminum content. Kumar J et al. [35] found that adding Al to the CoCrFeMnNi high-entropy alloy induces lattice distortion and the precipitation of secondary phases within the matrix of the CoCrFeMnNi high-entropy alloy, resulting in an increase in material hardness. J.Y. He et al. [37] found that the phase composition of the alloy changes with variations in the Al content. This suggests that laminated composites consisting of alternating layers of CoCrFeMnNi high-entropy alloy and aluminum intermetallic compounds are both feasible and adjustable in performance. This study primarily investigates the interfacial reactions and the evolution of interfacial morphology between these two components. Therefore, it is very necessary that the diffusion mechanism and evolution of intermetallic compounds in the interface need to be clarified and uncovered.

Herein, in the present study, the diffusion couple of CoCrFeMnNi HEA and pure aluminum was prepared. Current research on intermetallic compounds formed by reactions between CoCrFeMnNi high-entropy alloys and Al is limited. The required reaction temperature and duration for intermetallic compound formation during diffusion remain unclear. Existing studies [38,39] indicate that when low-melting-point Al reacts with higher-melting-point metals, the types and growth rates of resulting intermetallic compounds depend on the physical state of Al, with the melting point of Al being 660 °C. Therefore, the obtained diffusion couple is heated for 30 min, 60 min, 120 min and 240 min at 660 °C, 680 °C and 700 °C. Correspondingly, the microstructure evolution, formation mechanism, growth kinetics and growth thermodynamics of intermetallic compounds in different layers were investigated and clarified. Furthermore, the effects of temperature and holding time on the interdiffusion behavior were also investigated, and the activation energy of growth was also calculated. It is anticipated that this present study would provide a fundamental understanding and theoretical basis for CoCrFeMnNi HEA, potentially applied as a cast mold material for cast aluminum alloy.

## 2. Experimental Procedures

### 2.1. Sample Preparation

**Figure 1:**
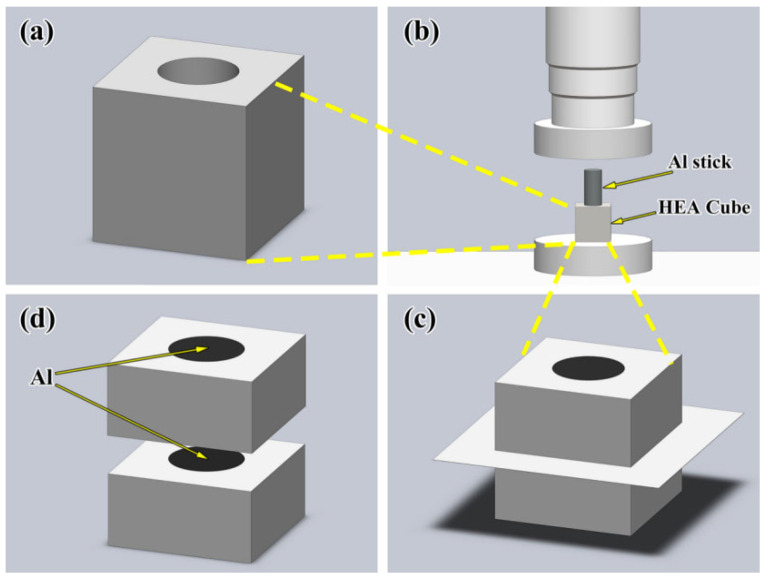
(**a**) CoCrFeMnNi cube with hole; (**b**) pressing the aluminum stick into the cube using compressor; (**c**) cutting from the cross-section of the couple; (**d**) CoCrFeMnNi/Al solid–liquid diffusion couple sample.

CoCrFeMnNi HEA ingot with equal atomic ratio is prepared by the vacuum medium-frequency induction melting, and all raw materials with the purity above 99.9 wt% are used. The CoCrFeMnNi HEA cubes, cut from the initial ingot by wire electric discharge machining (WEDM, Posittec, Suzhou, China), are drilled with a hole with a depth of 8 mm and a diameter of 4.6 mm from the middle of the surface, as shown in Figure 1a. Then, the cube is annealed at 1000 °C for 240 min, and the inner surface of the hole is ground by sandpapers from #800 to #2000. Industrial pure aluminum rod (99.9 wt%) with a diameter of 4.55 mm and a height of 11 mm, being polished to reduce the oxide layer, is pressed into the hole of the CoCrFeMnNi HEA cube by a compressor, as shown in Figure 1b. Subsequently, the obtained diffusion couple is heated for 30 min, 60 min, 120 min and 240 min at 660 °C, 680 °C and 700 °C, respectively, by ZT-25-20Y vacuum sintering furnace (SIMUWU, Shanghai, China) without gas, and all samples are cooled with the furnace to avoid the influence of cooling rate.

### 2.2. Microstructure Characterization

Samples cut from the cross section in the couples as shown in Figure 1d, are polished for microstructure examination using a field emission scanning electron microscope measurements employed a 20.0 kV accelerating voltage, 1.5 nA beam current and 8.5 mm working distance under 5 × 10^−6^ mbar vacuum, with EDS quantification conducted at 60 s live time, process time 5, and the working distance (WD) was fixed at 8.5 mm, the dead time was consistently maintained at 30–45% (<50% threshold) via pulse processor optimization (Fast mode enabled) and dynamic beam blanking, and output count rate stabilized at 22,000–28,000 cps (significantly exceeding the 5000 cps minimum requirement). Thin-foil specimens measuring 3 × 5 µm, prepared by focused ion beam (FIB, FEI Helios NanoLab G3, Hillsboro, OR, USA), are observed under the transmission electron microscopy (TEM, JEM-2100, JEOL, Tokyo, Japan). The average thickness of layers is calculated by multiple measurements by software.

## 3. Results and Discussion

### 3.1. Microstructure

**Figure 2:**
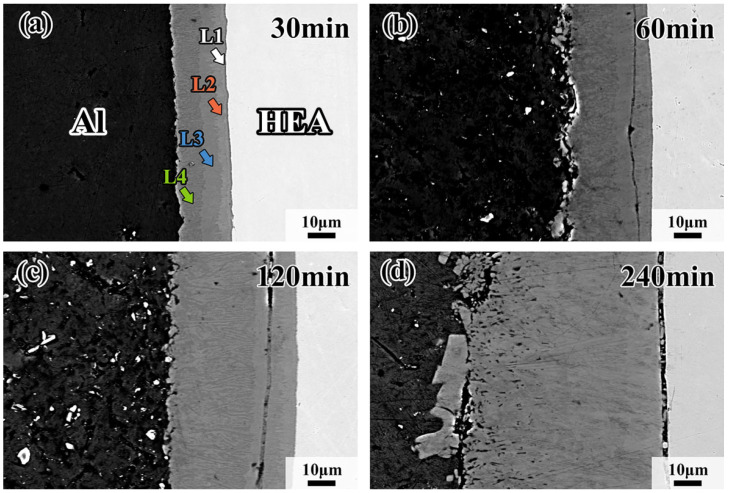
Morphologies of interfacial diffusion layer in CoCrFeMnNi/Al solid–liquid diffusion couples after annealing at 660 °C for (**a**) 30 min; (**b**) 60 min; (**c**) 120 min; (**d**) 240 min.

Figure 2 shows the microstructure of the interdiffusion area between CoCrFeMnNi HEA and Al after being treated at 660 °C for 30 min, 60 min, 120 min and 240 min. The average thickness of the interdiffusion area between the CoCrFeMnNi HEA and Al increases from 19 μm to 75 μm with the extension of holding time. It is clear there is a black ribbon between the HEA and L1 interfaces in Figure 2d. The black ribbon is the gap of HEA and the interdiffusion area. The formation of the gap is influenced by the different cooling rates of HEAs and the interdiffusion area. The interdiffusion area could be divided into four intermetallic compound layers, as revealed by different shades of color. For the convenience of the following description, the intermetallic compound layers in the interdiffusion area are named as L1, L2, L3 and L4 layers along the direction from the CoCrFeMnNi HEA to Al, shown in Figure 2a, respectively, and all layers (L1–L4) grow as continuous layers. Nevertheless, each layer presents a different morphology with increasing holding time. From Figure 2b–d, it can be found that the interface between L2 and L3 presents tentacle-like morphology, while the other layers are relatively straight. By quantitative analysis, the L4 layer accounts for the largest proportion, while the L1 layer is the smallest. In addition, with the increase in holding time, the thickness of the L4 layer significantly increases while the L1 layer increases slightly. When the holding time reaches 60 min, cracks can be found in the intermetallic compound layer near the CoCrFeMnNi HEA side and are mostly in the HEA/L1 interface or the L2 layer.

**Figure 3:**
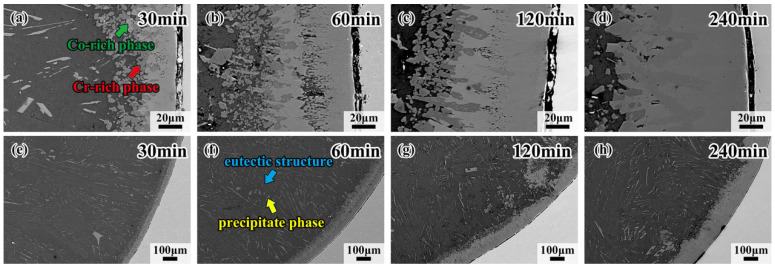
Morphologies of interfacial diffusion layers in CoCrFeMnNi/Al solid–liquid diffusion couples after diffusing at 680 °C for (**a**) 30 min; (**b**) 60 min; (**c**) 120 min; (**d**) 240 min; and internal precipitation phase of (**e**) 30 min; (**f**) 60 min; (**g**) 120 min; (**h**) 240 min.

**Figure 4:**
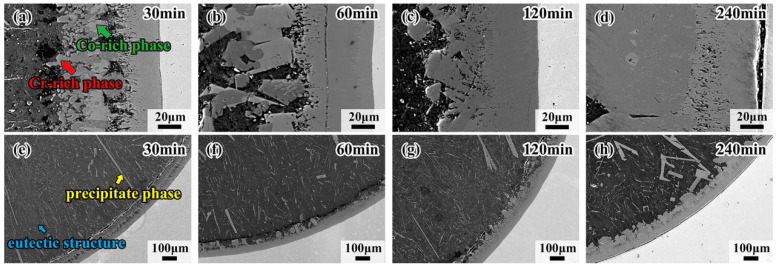
Morphologies of interfacial diffusion layer in CoCrFeMnNi/Al solid–liquid diffusion couples after annealing at 700 °C for (**a**) 30 min; (**b**) 60 min; (**c**) 120 min; (**d**) 240 min; and internal precipitation phase of (**e**) 30 min; (**f**) 60 min; (**g**) 120 min; (**h**) 240 min.

Figure 3 shows the morphology and detailed microstructure of the interfacial diffusion layer between the CoCrFeMnNi HEA and Al diffusion couple obtained after holding at 680 °C for 30 min, 60 min, 120 min and 240 min. There is the same black ribbon in samples like Figure 2d; the black ribbon is the gap between the HEA and the interdiffusion area. As can be observed in Figure 3a–d, the stripes in the L4 layer gradually change to blocks, demonstrating that the dark gray phase is doped in the bright gray phase. With the increase in holding time, the dark gray phase, trending to the Al side, grows from the blocks to thick strips. The bright gray phase approaching the CoCrFeMnNi HEA side grows into a whole sheet, forming the matrix of the dark gray phase. Note that the crack in the diffusion layer mostly occurs at the CoCrFeMnNi HEA/L1 interface, which should be attributed to the fact that the L1 layer is thinner than the other layers, and internal stress would thus be generated. It is also worth noting that the eutectic microstructure, in the form of short rods or thin strips, appears densely at the core of the Al side for the sample held for 30 min, as shown in Figure 3e. The precipitate phase and the eutectic structure appear together in the sample held at 680 °C for 60 min. The yellow arrows indicate the precipitate phases, which appear brighter due to their different contrasts. The blue arrows point to the dark gray matrix, which is aneutectic structure, shown in Figure 3f, and when the duration time increases to 240 min, the main phase in the Al core is solely the eutectic structure, which is shown in Figure 3h. Both the above precipitate phases grow toward the direction of the center of the circle as the holding time increases. Figure 4 shows the morphology of interfacial diffusion layers between CoCrFeMnNi HEA and Al diffusion couple being treated at 700 °C, holding for 30 min, 60 min, 120 min and 240 min. It is found that the dark gray phase grows partially until covering the whole L4 layer as the holding time increases from Figure 4a to Figure 4d. As can be seen from Figure 4e to Figure 4h, large strip phases with individual tissue up to 0.7 mm in length can be observed on the Al side, and the growing direction is towards the center of the circle.

### 3.2. Phase Composition of Diffusion Layer

**Figure 5:**
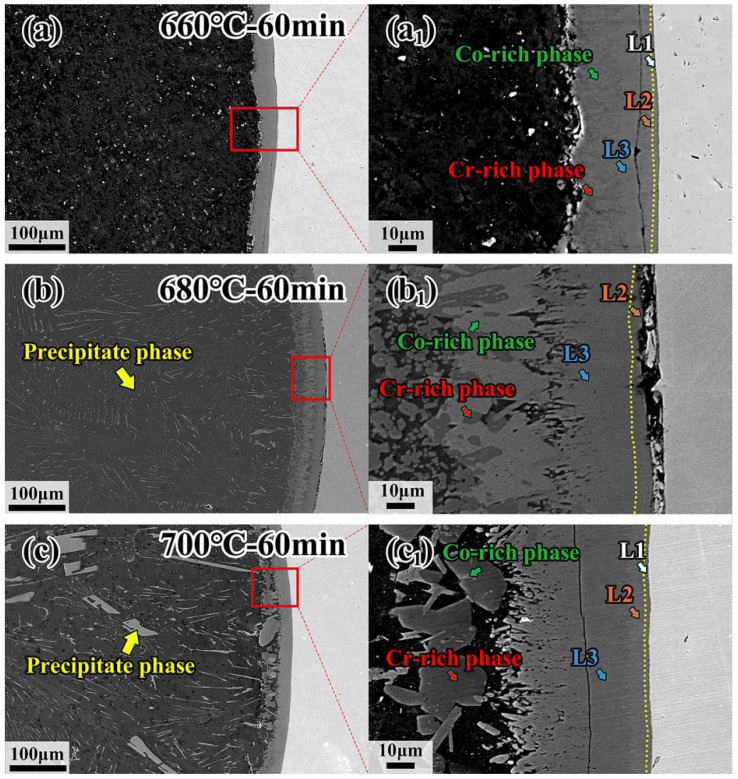
Microstructure of interfacial diffusion layer and internal precipitation phases in CoCrFeMnNi/Al solid–liquid diffusion couples after annealing at (**a**,**a_1_**) 660 °C—60 min; (**b**,**b_1_**) 680 °C—60 min; (**c**,**c_1_**) 700 °C—60 min.

**Figure 6:**
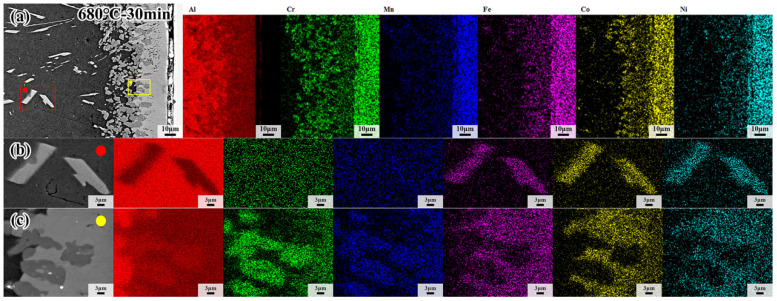
(**a**) EDS mapping results of CoCrFeMnNi/Al solid–liquid diffusion couple; (**b**) EDS mapping results of interfacial diffusion layer; (**c**) EDS mapping results of internal precipitation phase after annealing at 680 °C for 30 min.

The microstructure of CoCrFeMnNi HEA/Al diffusion couples treated at 660 °C, 680 °C and 700 °C for 60 min, and the specific process parameters are marked in Figure 5a–c. With increasing temperature, the precipitated phase becomes more visible, and the morphology of the precipitated phase in the center of the Al side changes from a block at low temperature to a strip at high temperature, as shown in Figure 5a–c. On the other hand, the thickness of the interdiffusion layer increases as the temperature increases, as shown in Figure 5a_1_–c_1_. However, the thickness of the interdiffusion layer in the sample held at 700 °C decreases due to the precipitated phase. The precipitated phase formed easily at high temperatures, and the atoms diffused into the Al core easily. Table 1 shows the EDS results by calculating the average of five points in each diffusion layer except for the L1 layer in Figure 5, and the EDS results of precipitated phases in the sample treated at 680 °C for 30 min are shown in Figure 6. It can be clearly seen from Figure 6 that the spherical dark gray phase is enriched in Cr and Mn elements, while the lamellar bright gray phase presents the enrichment of Fe, Co and Ni elements, and the intermetallic compounds in the Al core are rich in Fe, Co and Ni elements. The intermetallic compounds formed in a specified order, resulting from the different element properties, should be responsible for the enrichment of elements according to the EDS analysis. It has been demonstrated that the Al_5_Fe_2_ phase possesses the lowest phase formation enthalpy compared with other possible formation phases [40]. In this case, the Al_5_Fe_2_ phase would firstly come into being during diffusion, and subsequently a thin Al_13_Fe_4_ phase appears between the Al_5_Fe_2_ and Al [41,42,43,44]. It was noted that the formation of Al_5_Fe_2_ and Al_13_Fe_4_ phases is in the specific formation order. As a result, the Al_13_Fe_4_ phase appears first in the L2 layer. The constituent five elements in the present investigated CoCrFeMnNi HEA have very close atomic radii. In that regard, other elements could replace the position of the Fe element forming the Al_13_X_4_ (X = Co, Cr, Fe, Mn, Ni) type intermetallic compound in the L2 layer, as confirmed by EDS analysis.

In addition, interdiffusion between the CoCrFeMnNi HEA and Al becomes more predominant, and the width of the interdiffusion layer increases with the passage of time, thereby resulting in the formation of Al_4_Y (Y = Co, Cr, Fe, Mn, Ni) type phase in the L3 layer when the Al content approaches 80%, according to the binary phase diagram of Al-Cr and Al-Mn. As further confirmed by EDS analysis in Table 1, the content of the Al element is very close to the theoretical value, while the Co and Cr elements are enriched in the bright gray phase and dark gray phase in the L4 layer, respectively. This is consistent with the EDS surface scanning results in Figure 6. The compound in the L4 layer is Al_4_(Cr, Mn) phase in the dark gray area, while the Al_9_(Co, Fe, Ni)_2_ phase forms at the bright gray area. Since the activation of Cr and Mn elements is higher than other elements, the Al_4_(Cr, Mn) phase forms closely to the core of Al. The percentage of atomic number of Fe atoms, enhanced activity due to the higher temperature, increases slightly in the EDS results at the bright gray area, where the Al_9_(Co, Fe, Ni)_2_ forms, Co, Fe and Ni atoms assemble here because of their approachable atom radius and atom activation. It is inferred that L2 layer is Al_13_(Co, Cr, Fe, Mn, Ni) phase and L3 is Al_4_(Co, Cr, Fe, Mn, Ni) phase, while L4 consisting of Al_9_(Co, Fe, Ni)_2_ phase and Al_4_(Cr, Mn) phase and the Al core precipitated phase is the same as the Co-rich phase.

**Figure 7:**
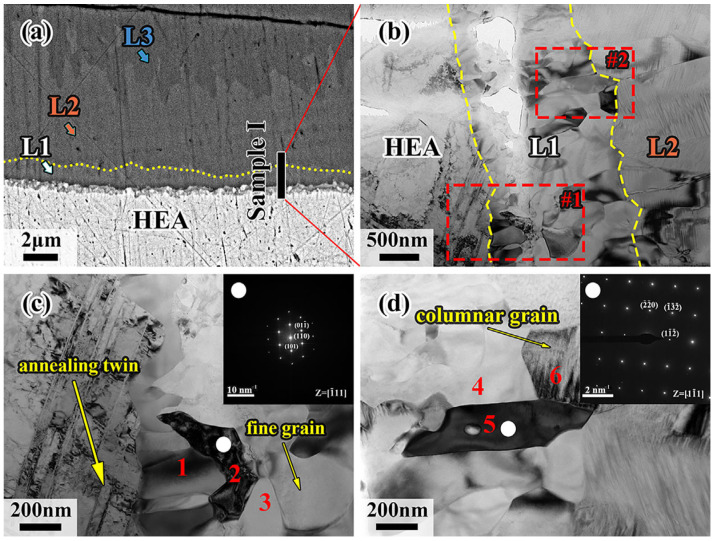
(**a**) Schematic diagram of FIB cutting positions of sample I; (**b**) morphology of sample I under TEM inspection; (**c**) higher magnification image of region #1; (**d**) higher magnification image of region #2.

Samples I, II and III, which were held at 700 °C for 120 min, cutting from the interdiffusion layer by the Focused Ion Beam Technique (FIB) due to the tiny size of the L1 layer, are represented as HEA-L1-L2, L2-L3, and L4, respectively. Figure 7c,d show enlarged images of regions 1 and 2 in Figure 7b. As can be seen in Figure 7c, annealed twins are found in the CoCrFeMnNi HEA by recrystallization during diffusion. However, fine crystals are generated at the CoCrFeMnNi HEA/L1 interface, and the L2 layer exhibits the coarse columnar crystals growing directionally. It was noted that the L1 layer is close to the CoCrFeMnNi HEA, and a relatively fast cooling rate and a large subcooling degree for crystallization would be induced. Additionally, a large amount of latent heat of crystallization would be released when the fine crystal zone forms, and it becomes difficult for liquid metal to dissipate heat and solidify due to the formation of interstices between solid CoCrFeMnNi HEA and liquid Al during solidification. Both of the above factors decrease the subcooling degree, making it difficult for nuclei to form. Eventually, the columnar grains grow perpendicularly due to the advantage in heat dissipation direction.

The microregion EDS results for the six positions in sample I are shown in Table 2, and the electron diffraction results for positions 2 and 5 are shown in Figure 7c,d. It is demonstrated that position 1 is the Cr-rich region, while position 3 is rich in Al and Ni elements according to the EDS results in Table 2. The elements of CoCrFeMnNi HEA in the two regions diffuse a considerable amount to the Al side, making the number of vacancies increase, which would locally be filled by the Al elements, forming a new phase. The CoCrFeMnNi HEA continuously discharges Ni atoms to form Al-Ni intermetallic compounds, and high energy in front of the diffusion layer makes Cr atoms, whose atomic radius is similar to Ni atoms, diffuse into Al, forming the Cr-rich phase. For position 2, the Al atom, possessing a larger atomic radius than that of the elements in the CoCrFeMnNi HEA, would cause the lattice distortions. Consequently, this results in the change of crystalline structure from FCC to BCC. This is confirmed by the content of the Al element in Table 2 and the SAED in Figure 7c. Figure 7d shows the SAED result of position 5 at the L1/L2 interface, where the diffraction pattern crystal band axis is [1$\overset{-}{1}$1], being close to the Al-Fe phase. The position of the Fe atom in the Al-Fe phase could be replaced by the other elements of the CoCrFeMnNi HEA, forming the Al(Co, Cr, Fe, Mn, Ni) phase based on the EDS results of positions 4~6, which shows great consistence with the previous conclusion. Therefore, the L1 layer can be divided into two regions: the Al-CoCrFeMnNi HEA diffusion transition layer near the CoCrFeMnNi HEA side and the Al(Co, Cr, Fe, Mn, Ni) phase near the L2 layer.

**Figure 8:**
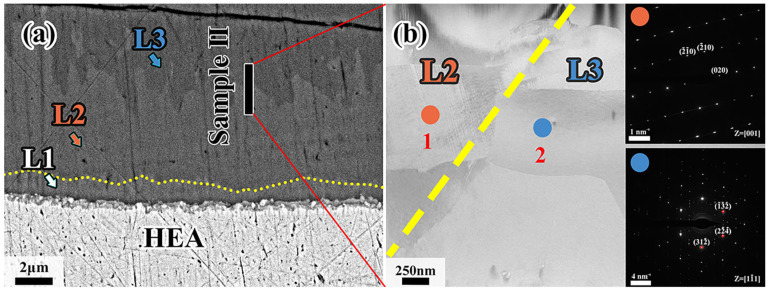
(**a**) Position of sample II in the diffusion layer; (**b**) higher magnification image of sample II and SAED of point 1, point 2 in L2 and L3, respectively.

Figure 8 shows the TEM results of sample II. It can be observed that sample II contains L2 and L3 diffusion layers whose grains are both columnar crystals. As further confirmed, the diffraction pattern at position 1 is identical to the Al_13_Co_4_ phase while position 2 coincides with the Al_4_Cr phase. The zone axes are reported [001] and [1$\overset{-}{1}$1], respectively. Therefore, it can be inferred that the L2 layer consists of Al_13_(Co, Cr, Fe, Mn, Ni)_4_ phase, while the intermetallic compound of the L3 layer is Al_4_(Co, Cr, Fe, Mn, Ni) phase, which coincides with the EDS results in Table 3.

**Figure 9:**
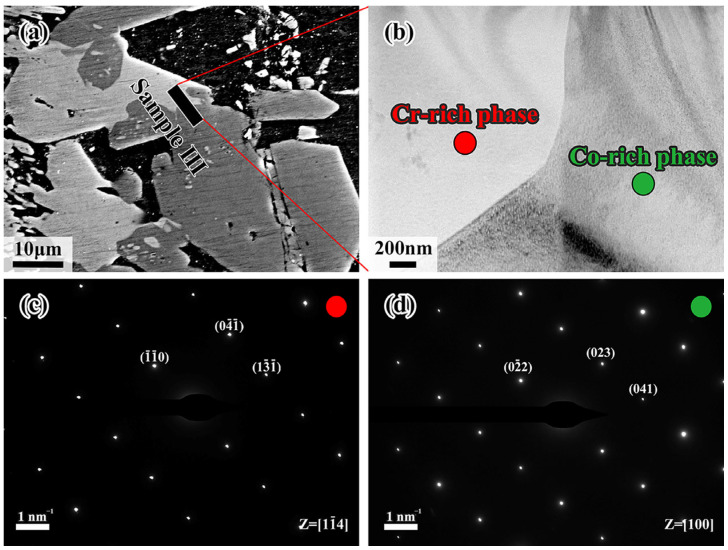
(**a**) Schematic diagram of the FIB cutting position for sample III; (**b**) TEM morphology of sample III; (**c**) SAED of point 1; (**d**) SAED of point 2.

Figure 9a shows the processing position of the FIB for sample III, using the same method as samples I and II. According to the EDS results in Table 4, sample III is composed of the Mn/Cr-rich region in the left and the Co/Fe/Ni-rich region in the right, which is consistent with the previous description. As identified by the SAED shown in Figure 9c,d, the red region, being rich in Cr element, corresponds to Al_4_Cr phase, while the green region enriched in Co element represents Al_9_Co_2_ phase, with the zone axis being [1$\overset{-}{1}$4] and [100], respectively. In that regard, the red and green regions are supposed to be the Al_4_(Cr, Mn) phase and Al_9_(Co, Fe, Ni)_2_ phase due to atomic substitution or occupation of vacancies, which clearly explains the presence of other elements by EDS analysis.

### 3.3. Diffusion Thermodynamics Analysis

#### 3.3.1. Diffusion Mechanism of Layers

In this study, the thickness of L2, L3 and L4 layers and the total thickness of the diffusion layer are statistically characterized for samples with various heat treatment conditions, respectively. The thickness is measured by Digital Micrograph software 3.21.1374.0, and the results are the average of at least ten measurements. In this study, the error bar, which is influenced by the comprehensive effect of coarse interface and measurement error, is not used. Due to the influence of cracks, the thickness of the L2 and L3 layers is statistically characterized only for samples treated at 680 °C and held for 60 min, 120 min and 240 min.

**Figure 10:**
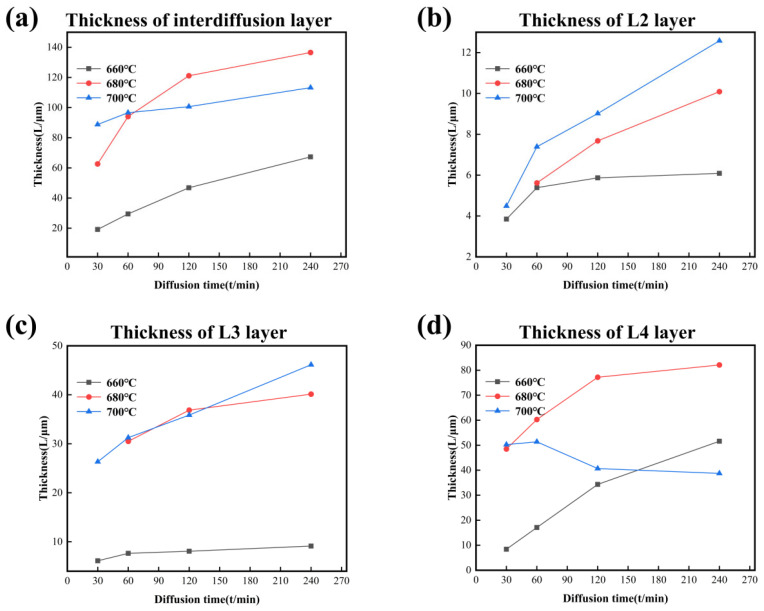
The thickness–diffusion time curves of IMCs layers form at different temperatures in CoCrFeMnNi/Al solid–liquid diffusion couples. (**a**) Interdiffusion layer; (**b**) L2 layer; (**c**) L3 layer; (**d**) L4 layer.

Figure 10 shows the thickness changes of the interdiffusion area and each layer with variation of temperature and time. The thickness of the interdiffusion area and each layer increases with time at all temperatures except the L4 layer, which decreases at 700 °C after the holding time reaches 60 min. The changes in thickness of the L4 layer indicate that elemental precipitation of CoCrFeMnNi HEA in the Al core occurs when the holding time is longer at 700 °C. In dynamics, the increased vibration frequency expands the atom’s ability to detach the bondage at the balance position due to the more energy at high temperatures. Meanwhile, the diffusion of atoms needs enough time to reach the core of Al. Under the comprehensive effect, the change discipline of thickness in the L4 layer after 60 min is abnormal compared to other layers. In addition, temperature is the main factor influencing the growth of the interdiffusion area because the growth rate and diffusion time are inversely proportional, especially at 680 °C and 700 °C. This viewpoint could be evidenced by the phenomenon that the thickness of the interdiffusion area or single layer was minimum at 660 °C.

According to previous studies, the thickness of the intermetallic compound layer, depending on diffusion time, can be expressed by the following equation [45]:

(1) $${L=kt}^{n}$$

where *L* is the thickness, t indicates the diffusion time, *k* represents the growth rate constant and n is a reaction kinetic index. In that regard, the growth mechanism of the intermetallic compound layer is determined by the value of *n*. It has been recognized that the value of n for the grain boundary diffusion mechanism is 0.25 [46], and diffusion proceeds through the grain boundary, where the defects are more numerous, such as vacancies.

The number of grain boundaries commonly makes a difference in the growing process of compounds at low temperatures or at the early diffusion stage. The correlation between the layer thickness and diffusion becomes linear as the value of n increases to 1, and then the compound’s growth is dominated by interfacial reaction, including solute redistribution at the interface or the degree of atomic mismatch. However, in practice, the growth mechanism of intermetallic compounds commonly involves a mixture of multiple mechanisms. For n values between 0.25 and 0.5, the bulk diffusion and grain boundary diffusion jointly dominate the compound’s growth. While for n values between 0.5 and 1, the growth of compounds is governed by both bulk diffusion and interfacial reaction.

**Figure 11:**
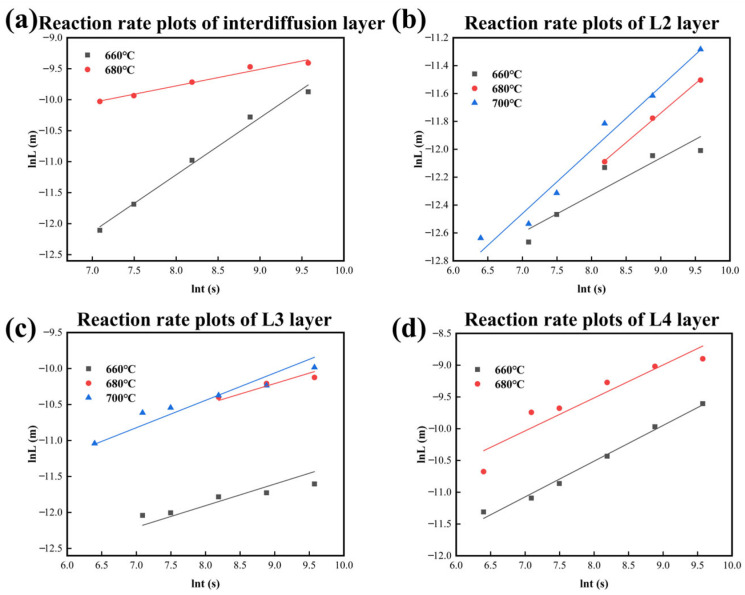
Reaction rate plots of IMCs layers form at different temperatures in CoCrFeMnNi/Al solid–liquid diffusion couples. (**a**) Interdiffusion layer; (**b**) L2 layer; (**c**) L3 layer; (**d**) L4 layer.

Figure 11 and Table 5 show the growth kinetic curves and n values for each layer, respectively, in which the L4 layer and the total diffusion layer at 700 °C are not included. It is noted that the value of n calculated by Equation (1) cannot accurately reflect the growth mechanism of the L4 layer because the redissolution occurs at 700 °C in this layer. It is ascertained that the values of n range around 0.5 at 660 °C and 680 °C, indicating that the bulk diffusion mechanism controls the growth of the total diffusion layer. For the diffusion process at 660 °C, the L2 layer forms at the early stage with an n value close to 0.25, and the growth of this layer is affected by the grain boundary diffusion mechanism. However, the L3 Layer is governed by mixed growth mechanisms, which involve grain boundary diffusion and bulk diffusion, with n values ranging from 0.25 to 0.5. The growth mechanism of the L4 layer is an interfacial reaction mechanism with the n value closing to 1. For the diffusion process at the other two temperatures (680 °C and 700 °C), the growth is dominated by mixed mechanisms combining grain boundary diffusion and bulk diffusion, with growth kinetic index n being in the range of 0.25 to 0.5. However, it is worth noting that the L4 layer forms as a particle precipitation phase rather than a continuous diffusion layer. In addition, the L4 layer not only grows transversely to a blocky tissue but also grows longitudinally, consequently slowing down the growth rate of the layer thickness and affecting the n value as the holding time increases.

#### 3.3.2. Activation Energy of Layer Growth

Activation energy of growth is the minimum energy required for layers to grow during diffusion. The Arrhenius equation of Equation (2) is used in the isothermal diffusion to express the activation energy of intermetallic layer growth as a function of temperature [47]:

(2) $$k^{2}=Aexp(-\frac{E_{a}}{RT})$$

where *k* is the growth rate constant, *A* is the exponential factor, *E_a_* is the activation energy of compound layer growth, *T* is the diffusion temperature and *R* is the gas constant. In that regard, the activation energy can be accordingly calculated for layers whose growth mechanism is grain boundary diffusion or a bulk diffusion mechanism, such as the L2 and L3 layers.

For the diffusion layer that grows by an interfacial reaction mechanism, such as the L4 layer, the energy required for the redistribution of solute atoms at the interface and the formation of a new phase can be calculated accordingly, instead of using the activation energy of growth. Therefore, this section only calculates the activation energy of growth in the L2 and L3 layers during the diffusion process.

**Figure 12:**
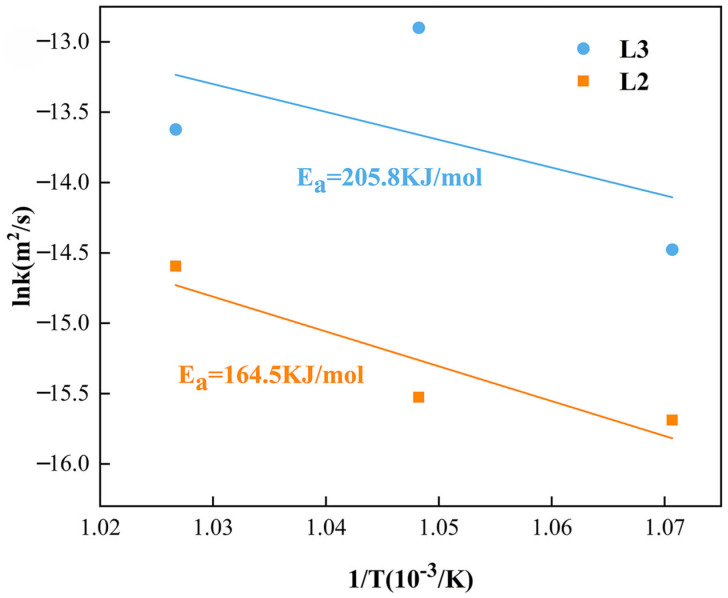
The logarithm of growth rate constants of IMCs layers versus inverse temperature.

By converting and plotting, the slope of the line fitted by linear regression can be determined, which is the activation energy of the compound layer. Figure 12 shows the curves of the intermetallic compound layers’ growth rate constant versus the inverse absolute temperature. In diffusion couples, the activation energy of Al_13_(Co, Cr, Fe, Mn, Ni)_4_ in the L2 layer is 164.5 KJ/mol and 205.8 KJ/mol for Al_4_(Co, Cr, Fe, Mn, Ni) in the L3 layer.

## 4. Conclusions

In the present study, the CoCrFeMnNi HEA/Al diffusion couple with different diffusion treatment conditions is prepared by hot-pressed sintering, aiming to reveal its interfacial microstructure evolution and interdiffusion behavior. The microstructure evolution of intermetallic compounds in the different interdiffusion layers, the formation mechanism and the activation energy of the interdiffusion area are investigated and clarified. The relevant results are summarized as follows:

1. At 660 °C, the intermetallic compounds controlled by temperature consist of a two-phase layer and a homogeneous layer, which form as continuous strips between the CoCrFeMnNi HEA and Al in the interdiffusion area, with no precipitations in the core of Al. The particle and block precipitations form in the two-phase layer after increasing the temperature.
2. In the order from the CoCrFeMnNi HEA to Al, the phases of the L1 to L3 layers, called the homogeneous layers, are Al(Co, Cr, Fe, Mn, Ni), Al_13_(Co, Cr, Fe, Mn, Ni)_4_ and Al_4_(Co, Cr, Fe, Mn, Ni), while the L4 layer, called the two-phases layer consisted of Mn/Cr-rich and Co/Fe/Ni-rich phases, namely Al_4_(Cr, Mn) and Al_9_(Co, Fe, Ni)_2_, respectively.
3. The growth mechanism of Al_13_(Co, Cr, Fe, Mn, Ni)_4_ and Al_4_(Co, Cr, Fe, Mn, Ni) is a mixture of grain boundary diffusion and bulk diffusion, while the growth of Al_4_(Cr, Mn) and Al_9_(Co, Fe, Ni)_2_ is controlled by bulk diffusion and interfacial reactions.
4. The slow diffusion effect of CoCrFeMnNi HEA causes a higher activation energy than other compounds with the same structure, which causes the higher activation energy of Al_13_(Co, Cr, Fe, Mn, Ni)_4_ and Al_4_(Co, Cr, Fe, Mn, Ni), which are 164.5 KJ/mol and 205.8 KJ/mol, respectively.

## Figures and Tables

**Table 1:** EDS analysis of the intermetallic phases form in solid–liquid diffusion.

Sintering Parameters	Region	Content (at.%)					
		Al	Cr	Mn	Fe	Co	Ni
660 °C—60 min	L2	77.48	4.96	4.55	4.36	4.51	4.14
	L3	82.80	4.32	4.53	3.18	2.37	2.80
	Co-rich	83.68	0.68	0.83	3.58	5.66	5.57
	Cr-rich	84.97	5.30	5.66	1.68	0.86	1.53
680 °C—60 min	L2	79.49	4.12	4.80	3.85	3.99	3.75
	L3	80.84	4.36	4.32	4.11	3.57	2.80
	Co-rich	83.03	1.07	0.72	4.21	8.10	2.87
	Cr-rich	86.87	8.14	2.79	0.85	0.63	0.72
	Precipitate phase	82.96	0.09	0.37	4.95	7.64	3.99
700 °C—60 min	L2	77.83	4.84	3.67	4.87	4.79	4.00
	L3	81.08	4.62	4.32	4.54	3.40	2.04
	Co-rich	82.62	0.41	0.36	4.19	9.50	2.92
	Cr-rich	82.73	7.91	4.73	2.25	1.34	1.04
	Precipitate phase	82.68	0.19	0.50	4.65	8.00	3.98

**Table 2:** EDS analysis of sample I.

Region	Content (at.%)					
	Al	Cr	Mn	Fe	Co	Ni
1	0.24	33.37	20.42	27.50	14.80	3.65
2	19.93	20.15	14.86	7.44	11.29	26.30
3	25.86	1.08	10.73	4.59	20.58	37.12
4	43.64	13.16	9.32	14.68	11.85	7.31
5	44.94	10.24	10.50	13.76	13.92	6.60
6	51.80	7.79	8.27	9.67	14.01	8.42

**Table 3:** EDS analysis of sample II.

Region	Content(at.%)					
	Al	Cr	Mn	Fe	Co	Ni
1 (L2)	75.38	5.30	5.27	5.03	4.00	5.02
2 (L3)	79.26	5.71	5.07	3.12	3.39	3.45

**Table 4:** EDS analysis of sample III.

Region	Content (at.%)					
	Al	Cr	Mn	Fe	Co	Ni
Cr-rich	81.39	12.85	3.66	0.92	0.23	0.95
Co-rich	78.62	0.21	0.45	5.82	10.02	4.88

**Table 5:** Growth kinetic index of each IMC form at different temperatures.

Temperature (°C)	n			
	Total	L2	L3	L4
660	0.52	0.26	0.30	0.94
680	0.51	0.40	0.29	0.33
700	—	0.43	0.38	—

## Data Availability

The original contributions presented in this study are included in the article. Further inquiries can be directed to the corresponding authors.
